# Neoadjuvant Chemotherapy and Appleby Procedure for Pancreatic Acinar Cell Carcinoma: A Case Report

**DOI:** 10.1089/crpc.2016.0009

**Published:** 2016-06-01

**Authors:** Masaya Jimbo, Philip M. Batista, Jeffrey P. Baliff, Charles J. Yeo

**Affiliations:** ^1^ Department of Surgery, Jefferson Pancreas, Biliary, and Related Cancer Center, Thomas Jefferson University, Philadelphia, Pennsylvania.; ^2^ Department of Pathology, Anatomy, and Cell Biology, Thomas Jefferson University, Philadelphia, Pennsylvania.

**Keywords:** acinar cell carcinoma, Appleby procedure, neoadjuvant chemotherapy

## Abstract

**Background:** Acinar cell carcinoma is a rare form of pancreatic cancer, accounting for 1–2% of all cases of exocrine pancreatic neoplasms in adults. Due to its rarity, no randomized controlled trials have been performed to determine the optimal treatment options. As such, high-quality case reports and case series are needed to help guide clinicians in the management of this deadly disease.

**Case Presentation:** A 56-year-old Caucasian male presenting with abdominal pain and weight loss was diagnosed with stage III acinar cell carcinoma of the pancreatic body with celiac axis involvement. Although initially deemed unresectable, the patient responded favorably to nine cycles of 5-fluorouracil-based neoadjuvant chemotherapy. The tumor was successfully resected through distal pancreatectomy with *en bloc* splenectomy and *en bloc* celiac artery resection (Appleby procedure). Final pathology analysis showed negative resection margins and complete chemotherapeutic response within the pancreas, with residual tumor cells detected in only a single peripancreatic lymph node.

**Conclusion:** 5-fluorouracil-based chemotherapy may be a promising option for the neoadjuvant treatment of locally unresectable acinar cell carcinoma. With sufficient expertise, negative surgical resection margins are possible even with vascular involvement. Due to the generally poor prognosis associated with acinar cell carcinoma, such aggressive treatment measures are warranted.

## Introduction

Acinar cell carcinoma is a rare form of pancreatic cancer, accounting for 1–2% of all cases of exocrine pancreatic neoplasms in adults. Due to its rarity, data regarding the most effective treatment options for this disease are lacking. Herein, we report the case of a patient with initially unresectable, locally advanced acinar cell carcinoma with celiac artery involvement, who was successfully treated with the combination of neoadjuvant chemotherapy and the Appleby procedure.

## Case

**FIG. 1.**
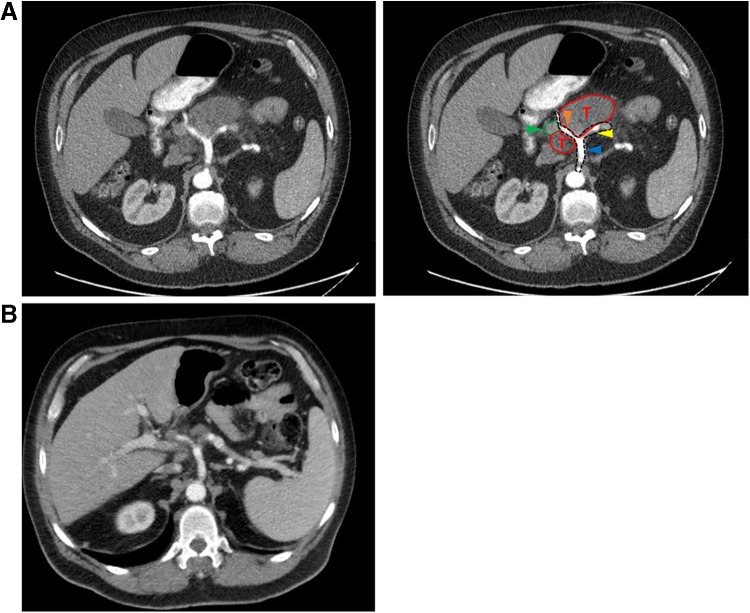
Computed tomography scans of the patient's abdomen, **(A)** before and **(B)** after nine cycles of combined chemotherapy. Blue arrowhead = celiac trunk. Yellow arrowhead = splenic artery. Orange arrowhead = common hepatic artery. Green arrowhead = portal vein. T, tumor.

A 56-year-old Caucasian male presented in September 2015 with severe, cramping, epigastric abdominal pain that radiated to his back and was associated with nausea, emesis, decreased appetite, and a 10 pound weight loss over the prior several months. Computed tomography (CT) of the abdomen (Fig. 1A) revealed a 6 cm mass in the body of the pancreas, closely adjacent to the celiac trunk, with main pancreatic duct dilation and parenchymal atrophy of the pancreatic tail. Although no signs of hepatic or distant metastatic disease were present, prominent regional celiac nodes were found. Fine needle aspiration pancreatic biopsy 2 days later detected cells compatible with acinar cell carcinoma. Per the AJCC TNM staging system, the patient was diagnosed with T4N1M0 (stage III) acinar cell carcinoma.^1^

The patient proceeded to receive neoadjuvant chemotherapy with FOLFIRINOX (folinic acid, 5-fluorouracil, irinotecan, and oxaliplatin), beginning in October 2015. He received three cycles with dose reduction secondary to side effects such as diarrhea. After the third cycle, he refused additional chemotherapy and requested evaluation at Thomas Jefferson University Hospital (TJUH) for possible surgical resection. A new CT scan was obtained, and although it showed a slight decrement in the size of the tumor, he was still deemed unresectable, and it was recommended that he continue chemotherapy.

The patient resumed chemotherapy in late December with 5-fluorouracil, leucovorin, and oxaliplatin (FOLFOX). The patient tolerated this regimen remarkably well and completed six such cycles by March 2016, for a total of nine cycles of neoadjuvant chemotherapy. A repeat CT scan of the abdomen (Fig. 1B) showed continued partial response, with the pancreatic body mass now measuring only 2.0 × 1.3 cm (vs. 3.1 × 2.0 cm in December) and the dominant celiac lymph node now measuring only 1.7 × 1.6 cm (vs. 2.5 × 2.3 cm in December). He was reevaluated at TJUH, and this time he was deemed suitable for attempted surgical resection. Notably, his serum levels of CA19-9 (11 U/mL), carcinoembryonic antigen (1.6 ng/mL), and lipase (15 U/L) were all within normal limits immediately before resection.

**FIG. 2.**
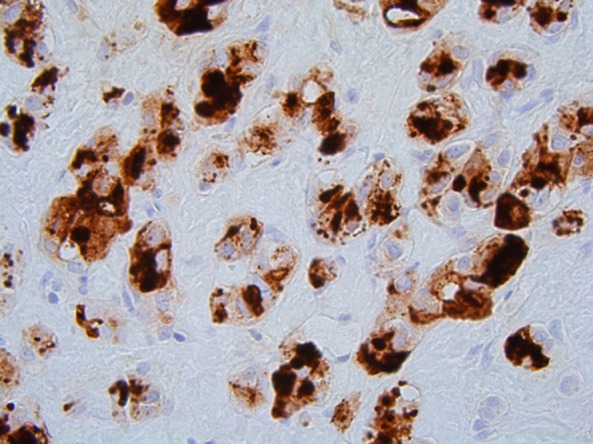
Trypsin immunohistochemical stain (400 × ). Peripancreatic lymph node shows strong positivity in trypsin-positive tumor cells, supporting acinar differentiation.

The operation was performed at TJUH on April 2016. Due to the involvement of the celiac axis, the patient underwent distal pancreatectomy with *en bloc* splenectomy and *en bloc* celiac artery resection (Appleby procedure). The patient tolerated the procedure well, the postoperative course was uncomplicated, and he was discharged on postoperative day 7. Final pathology analysis showed negative resection margins and extensive chemotherapy effect, with only a single 0.3 cm focus of residual grade 1 carcinoma in a large peripancreatic lymph node. No carcinoma was identified in the pancreatic body proper, indicating a complete response in the pancreas. The residual tumor cells in the lymph node were analyzed by immunohistochemistry (IHC). These cells showed positive staining for trypsin and negative staining for synaptophysin, chromogranin, and beta-catenin, strongly supporting the previous diagnosis of acinar cell carcinoma (Fig. 2).

## Discussion

Acinar cell carcinoma of the pancreas is quite rare, accounting for just 1–2% of exocrine pancreatic neoplasms in adults.^2–4^ It is predominantly found in older patients, with peak incidence in patients in their 60s (seventh decade of life), and it is twice as common in men compared with women.^2,5^ As was the case with our patient, presenting symptoms tend to be nonspecific and include anorexia, nausea, vomiting, and abdominal pain.^4^ In contrast to pancreatic ductal adenocarcinoma (PDA), jaundice is a rare finding in acinar cell carcinoma.^2,4^ The Schmid triad of subcutaneous fat necrosis, polyarthralgia, and eosinophilia is only seen in 15% of patients, and is due to lipase hypersecretion by the tumor.^3,4,6^ The classic genetic mutations associated with PDA, for example, the activation of *KRAS2* and inactivation of *p16/CDKN2A*, *TP53*, and *SMAD4/DPC4*, are very rarely seen.^7^ Instead, the mutation profile of acinar cell carcinoma is more comparable to that of pancreatoblastoma, with both tumor types frequently exhibiting allelic loss on chromosome 11p and alterations in the beta-catenin pathway (e.g., APC truncation and beta-catenin activation).^7^ IHC demonstrating acinar differentiation is essential for diagnosis. The diagnosis of acinar cell carcinoma is supported by positive staining for digestive enzymes such as trypsin, lipase, chymotrypsin, and amylase, as well as negative staining for neuroendocrine markers such as chromogranin A and synaptophysin.^2,7^ However, a subset of acinar cell carcinomas can have scattered foci of endocrine differentiation that stain positively for chromogranin A or synaptophysin.^4^

Although the prognosis for patients with acinar cell carcinoma is improved compared to patients with PDA, it is still very poor, with median survivals for patients with localized and metastatic disease reported to be 38 and 14 months, respectively.^8^ Surgical resection provides the best outcome (median survival of 36 months in patients who undergo surgery compared with 14 months in patients who do not undergo surgery); however, the recurrence rate is very high (72%), and the 5-year survival is <6%.^4,8,9^

Acinar cell carcinoma is managed with the same surgical procedures used to manage PDA. Depending on the size and localization of the tumor, these procedures include pancreaticoduodenectomy (Whipple procedure), distal pancreatectomy, and total pancreatectomy.^5^ Tumors in the pancreatic body and tail are often diagnosed with involvement of the nearby celiac artery, and such tumors have generally been deemed unresectable.^10^ However, in certain circumstances, the modified Appleby procedure, which consists of distal pancreatectomy with *en bloc* splenectomy and celiac artery resection, can be performed to achieve negative resection margins in patients with such tumors.^10^ This procedure requires adequate collateral circulation between the superior mesenteric artery and the proper hepatic artery (PHA) through the gastroduodenal artery (GDA) to prevent catastrophic hepatic ischemia after resection of the celiac artery and associated common hepatic artery (CHA).^10^ Our patient presented with acinar cell carcinoma that originated in the pancreatic body and encased the celiac artery. Upon intraoperative clamping of the CHA, a strong Doppler signal pulse was detected within the PHA. This suggested the presence of adequate GDA collaterals, and as such the modified Appleby procedure could be performed to achieve a margin-negative resection.

Roughly half of acinar cell carcinoma patients present with metastatic or locally unresectable disease.^4^ Treatment guidelines for these patients have not been established, as published reports are limited to case reports and case series. In the absence of data from prospective, randomized controlled trials, systemic therapy for these patients usually incorporates chemotherapy agents with known efficacy against PDA or colorectal carcinoma (CRC), the latter because a subset of acinar cell carcinomas exhibits the same mutations in the beta-catenin pathway as CRC.^4,11^ A 2011 case series of patients with acinar cell carcinoma reported partial response to gemcitabine-based or 5-fluorouracil-based combination chemotherapy in 30% of patients with metastatic disease, and suggested that these combination regimens may offer significant activity against acinar cell carcinoma.^11^ Indeed, our patient was treated with 5-fluorouracil-based combination therapy (three cycles of FOLFIRINOX followed by six cycles of FOLFOX). Not only did the treatment show sufficient antitumor response to render an initially unresectable tumor ultimately resectable with negative surgical margins, but the final pathology analysis also showed extensive chemotherapy effect, with residual carcinoma undetectable within the pancreas specimen.

## Conclusion

To our knowledge, this is the first case report of locally advanced acinar cell carcinoma of the pancreas that was successfully managed by neoadjuvant combination chemotherapy followed by the Appleby procedure. The obliteration of all carcinoma within the pancreatic body (a complete response) suggests that 5-fluorouracil-based regimens employed in PDA treatment (FOLFIRINOX and FOLFOX) may be highly effective for acinar cell carcinoma as well. Even if combination chemotherapy is insufficient to shrink the tumor enough for classic pancreaticoduodenectomy or distal pancreatectomy, in certain conditions alternative methods such as the Appleby procedure can be used to achieve margin-negative surgical resection. Given the poor prognosis of patients with acinar cell carcinoma without surgical resection, aggressive treatment approaches are warranted to maximize their chance for survival.

## Abbreviations Used

CHA: common hepatic artery
CRC: colorectal carcinoma
CT: computed tomography
GDA: gastroduodenal artery
IHC: immunohistochemistry
PDA: pancreatic ductal adenocarcinoma
PHA: proper hepatic artery
